# Racial discrimination is associated with food insecurity, stress, and worse physical health among college students

**DOI:** 10.1186/s12889-024-18240-3

**Published:** 2024-03-22

**Authors:** Ryan Gamba, Negin Toosi, Lana Wood, Alexandra Correia, Nomar Medina, Maria Pritchard, Jhamon Venerable, Mikayla Lee, Joshua Kier Adrian Santillan

**Affiliations:** 1 Department of Public Health, California State University, East Bay. SF 102. 25800 Carlos Bee Boulevard, 94542 Hayward, CA USA; 2 Department of Psychology, California State University, East Bay. 25800 Carlos Bee Boulevard, 94542 Hayward, CA USA; 3 University Libraries, California State University, East Bay, 94542 Hayward, CA USA

**Keywords:** Racial discrimination, Food insecurity, College students, Police discrimination

## Abstract

**Background:**

Students of color disproportionately experience racial discrimination and food insecurity, which both lead to poor academic and health outcomes. This study explores the extent to which the location of racial discrimination experienced is associated with food insecurity, stress, physical health and grade point average among college students

**Methods:**

A cross sectional study design was implemented to survey 143 students from a racially diverse public university. Logistic regression models assessed if discrimination at various locations was associated with food insecurity and linear models assessed how racial discrimination was associated with physical health, stress and grade point average

**Results:**

Student’s experiencing food security had an average discrimination score of 2.3 (1.23, 3.37), while those experiencing food insecurity had a statistically significant (*P* < 0.001) higher average discrimination score 7.3 (5.4, 9.21). Experiencing any racial discrimination was associated with increased odds of experiencing food insecurity when experienced from the police (OR 11.76, 95% CI: 1.41, 97.86), in the housing process (OR 7.9, 95% CI: 1.93, 32.34) and in the hiring process (OR 6.81, 95% CI: 1.98, 23.48) compared to those experiencing no racial discrimination after adjusting for race, gender, age and income.

**Conclusion:**

The location in which a student experienced racial discrimination impacted the extent to which the racial discrimination was associated with food security status. Further research is needed to explore potential mechanisms for how racial discrimination may lead to food insecurity.

## Introduction

Research suggests racial discrimination, or the unfair or prejudicial treatment of a person or group of people on the basis of their group membership, [1] is experienced by students of all racial/ethnic backgrounds and disproportionately among communities of color. Racial discrimination among college students has been linked to poor health outcomes such as anxiety, depression, suicidal ideation, [2, 3] and in some cases, poorer academics [4–6].

Similarly, food insecurity, which is indicated “whenever the availability of nutritionally adequate and safe food, or the ability to acquire acceptable foods in socially acceptable ways, is limited or uncertain” [7] is also disproportionately experienced by students of color [8] and associated with poor health and academic outcomes [9, 10]. Among college students, experiencing food insecurity has been associated with a 0.09 lower grade point average after adjusting of covariates [11], and a 2.29 greater odds of reporting fair/poor health compared to those who are food secure [12]. Despite a large increase in food pantries opening up across college campuses nationwide [13], the prevalence of food insecurity among college students consistently exceeds that of the national average [14].

Researchers have recently explored racism and racial discrimination as potential causes of food insecurity [15–18]. While persistent racial disparities in the prevalence of food insecurity are often attributed to differences in income and poverty, it is clear that there is a positive association between racial discrimination and food insecurity after adjusting for differences in socioeconomic status. Among Latinx mothers, Phojanakong et al. found that experiencing discrimination in school was associated with 1.58 (95% CI: 1.05–3.26) higher odds of experiencing household food insecurity and a 1.79 (95% CI: 1.12–2.86) higher odds of depressive symptoms compared to those who did not face discrimination [16]. Dombrowski suggests that components of historical structural racism, such as land grabs, red-lining, and access to healthcare, have influenced components of modern structural racism such as inequitable food environments [18]. Bowen cites Phelan et al.’s “fundamental cause theory,” and states racism is a fundamental cause of food insecurity, meaning food insecurity cannot be fully addressed without addressing racism and racial disparities [15]. While these works have focused on structural racism, racial discrimination has also been identified as a possible cause of food insecurity [16, 17, 19].

Despite the co-occurrence of racial discrimination and food insecurity among college students, few studies have assessed the relationship between racial discrimination and food insecurity in this population. Mahoney et al. recently showed all-cause discrimination led to food insecurity among LatinX college students [17]. Among non-college students, Phojanakong et al. showed that experiencing discrimination in particular aspects of one’s life (ex. At the workplace, in the hiring process) were significantly associated with increased odds of food insecurity while discrimination experienced elsewhere was not [16]. No studies have been identified that examine if the location of where the racial discrimination occurred (in the classroom, applying for housing, etc.) is an important consideration in the racial discrimination and food insecurity relationship among college students.

Phelan and Link revisited the model of social conditions as fundamental causes of disease and suggested not only does racism act through socioeconomic status to impact health disparities, but racism has an independent fundamental impact on health and health disparities [20]. Citing racial differences in power, prestige, freedom, neighborhood context, and healthcare, it is argued that racism limits access to resources that can prevent disease. Similarly, studies suggest that racism is a fundamental cause of the academic achievement gap, where racism may inhibit students from accessing the resources they need to succeed in the classroom [21]. Given racial discrimination is argued to be a fundamental cause of both health and academics, we hope to build upon past research that has largely focused on racial discrimination and mental health outcomes of students [2, 3], and studies that have investigated racial discrimination and academics and have found mixed results [4–6].

This study has two aims; First, to investigate how experiencing racial discrimination in a particular aspect of one’s life is associated with food insecurity in a racially diverse sample of college students; and second, to explore in this sample the extent to which a composite score of racial discrimination is associated with food insecurity, stress, physical health, and grade point average.

## Methods

Students form a diverse public university were recruited via flyers, emails and class announcements (across an array of disciplines) to take surveys between March 9, 2021 and May 18, 2022. Participants were eligible if they were currently enrolled at the university and over 18 years of age. Of the 181 surveys, those with missing information for income (*n* = 2), race (*n* = 12), gender (*n* = 2), physical health (*n* = 4), stress (*n* = 3) and food insecurity (*n* = 18) were not included in the study. Our final analytical sample was 143 participants, as some participants were missing more than one piece of information.

### Independent variable– racial discrimination

To measure racial discrimination, we used the Experiences of Discrimination scale developed by Krieger and colleagues (2005) [22]. Participants were asked if they had ever “experienced discrimination, been prevented from doing something, or been hassled or made to feel inferior” because of racial bias in any of a number of situations. The list of the situations were as follows: at school, getting hired or getting a job, at work, getting housing, getting medical care, getting service in a store or restaurant, getting credit, bank loans, or a mortgage, on the street or in a public setting, and from the police or in the courts. For each situational component, participants responded on a scale which, in accordance with Krieger et al. ‘s frequency coding, we marked as follows: never (0), once [1], two to three times (2.5) or four or more times [5]. The Racial Discrimination composite score was coded both continuously using this frequency coding (mean: 5.59, sd 8.82), and binary (experienced some discrimination, experienced no discrimination). Each component of the composite score was recorded as a binary variable (experienced some discrimination, experienced no discrimination).

### Dependent variables– physical health, stress, grade point average and food insecurity

#### Physical health

Physical health was measured using six items adapted from Ware and colleagues’ (1996) Short-Form Health Survey [23]. One item, “In general, how is your health?” (reverse-scored) used a response scale from 0 (*very bad*) to 4 (*excellent*). The remaining five items, e.g. “In the past month, how often have you felt dizzy or fainted?” and “In the past month, how often has your physical health prevented you from accomplishing as much as you would like?” were answered on a response scale from 0 (*never*) to 4 (*very often*). The responses to these questions were averaged (mean 2.28, sd: 0.81) and coded as a continuous variable.

#### Stress

A 10-item measure of Perceived Stress was used to measure stress [24]. This measure used a 5-point scale with anchors at 0 (*never*) to 4 (*very often*). Sample items include “In the last month, how often have you felt that you were unable to control the important things in your life?”, “In the last month, how often have you found that you could not cope with all the things that you had to do?” and “In the last month, how often have you felt that things were going your way?” (reverse-scored). The responses to these questions were averaged (mean 2.14, sd: 0.58) and coded as a continuous variable.

#### Academic performance (GPA)

Grade point average was self-reported and coded categorically as students with an A average, B average, or C average. No students reported a GPA that indicated a D or F average.

#### Food insecurity

The 10 item Household Food Security Survey Module was used to assess food security status [25]. Food security status was reduced to a binary indicator, where students who indicated affirmatively to one or more food insecurity questions. Often those who are experiencing marginal food security are combined with those with full food security to create a food secure category, however past research suggests that those experiencing marginal food security have significant differences than those that are food secure [26, 27]. While ideally this would be its own category, to avoid small cells and positivity violations we implemented a similar approach to past research where those experiencing marginal food security were combined with those who were considering low or very low food insecurity to create a food insecure group.

### Covariates

Race was self-reported. Participants indicating that they were Black (*n* = 5) or Native American (*n* = 2) were included in those with the Other Race category. Those indicating they were South Asian (*n* = 3), Middle Eastern or North African (*n* = 6) or Vietnamese (*n* = 6) were considered Asian Not previously listed. When groups had 10 or more individuals, we created a separate category for them as individuals’ racial discrimination experiences are heterogenous among normally implemented socially constructed groups such as Asian. Income (<$30,000, $30,000 to $49,999, $50,000 to $69,999, $70,000 to $89,999, and >$90,000), gender (male, female), and age were also coded categorically (18–21, 22–25, 26–29, >= 30, or missing). Age was the only variable coded that included a category for individuals who were missing their age information (*n* = 16) and this was done to preserve sample size.

### Statistical analysis

Linear regressions were implemented to determine if average discrimination scores significantly varied across food security status, grade point average and the other covariates. The relationship between individual components of the discrimination scale and the odds of food insecurity were explored with logistic regressions where adjusted models included controlling for race, gender, age and income. The relationship between discrimination and physical health, stress, grade point average, and food insecurity were explored when discrimination was coded both as a continuous variable, and as a binary variable where (discrimination experienced yes vs. no). Linear regression models were implemented to assess the association between discrimination and stress and physical health. Logistic models assessed the association between discrimination and food insecurity, and ordered logistic regression models assessed the associations between discrimination and grade point average. All of these models adjusted for race, gender, age and income. All analyses were conducted in Stata (version 17.0, StataCorp, College Station, TX). Standard errors were calculated with Stata’s VCE (robust) command. California State University, East Bay’s Institutional Review Board approved this study.

## Results

The student population was composed of predominantly females aged 18–25 who reported strong academic standing, where 45% reported an A or A- grade point average (Table 1). The average discrimination score was 5.59 (4.23, 6.95), and discrimination scores were significantly higher among Asian’s not previously listed (previously listed included Chinese, Filipino and Asian Indian), and among those who were classified in the Other Race category, compared to White students (*P* < 0.05). Student’s experiencing food security had an average discrimination score of 2.3 (1.23, 3.37), while those experiencing food insecurity had a statistically significant (*P* < 0.001) higher average discrimination score 7.3 (5.4, 9.21).

**Table 1:** Average discrimination scores across population characteristics among students (n = 143)

Covariate	n	(Proportion of population)	Average Discrimination Score^a^
**Gender**			
Male (ref)	29	20.28	3.78 (1.87, 5.68)
Female	114	79.72	6.04 (4.42, 7.67)
**Race**			
White (ref)	16	11.19	2.5 (0.62, 4.38)
Chinese	10	6.99	5.95 (0.77, 11.13)
Filipino	12	8.39	4.70 (1.53, 8.06)
Asian Indian	12	8.39	5.54 (2.73, 8.35)
Asian not previously listed	27	18.88	7.94 (3.68, 12.21)*
Other Race	29	20.28	8.26 (4.68, 11.83)*
Mixed Race	15	10.49	4.37 (-0.27, 9.00)
Latinx	22	15.38	2.54 (0.92, 4.15)
**Income**			
< $30,000 (ref)	51	35.66	5.83 (3.39, 8.28)
$30,000 to $49,999	24	16.78	9.29 (4.24, 14.33)
$50,000 to $69,999	27	18.88	3.52 (1.75, 5.29)
$70,000 to $89,999	18	12.59	4.42 (2.49, 6.34)
>$90,000	23	16.08	4.52 (2.14, 6.90)
**Food Security Status**			
Food Secure (ref)	49	34.27	2.30 (1.23, 3.37)
Food Insecure	94	65.73	7.30 (5.40, 9.21)***
**Grade Point Average**			
A/A-	65	45.45	6.15 (3.96, 8.33)
B +,B, B-	68	47.55	5.65 (3.73, 7.56)
C+, C, C-	10	6.99	1.55 (-0.9, 3.19)
**Age**			
18–21 (ref)	44	30.77	5.65 (3.22, 8.07)
22–25	51	35.66	4.35 (2.31, 6.40)
26–29	17	11.89	6.97 (2.31, 11.63)
>=30	15	10.49	10.30 (5.05, 15.55)
Missing	16	11.19	3.47 (0.60, 6.34)
Age	Average: 24.38 (23.89, 24.88)	100	5.59 (4.23, 6.95)

^a^Linear regressions were implemented to assess if the average discrimination scores were significantly different than the reference group at *P < 0.05, **P < 0.01, ***P < 0.001

Table 2 shows the relationship between individual components of the racial discrimination scale and food insecurity. The proportion of students reporting racial discrimination varied from 13.99% (*n* = 20) for those who experienced racial discrimination when trying to acquire a loan to 39.86% (*n* = 57) of students who experienced racial discrimination in a restaurant. In models adjusted for race, gender, age and income, racial discrimination was associated with a significantly increased odds of food insecurity when the discrimination was experienced at school, getting housing, getting medical care, on the street, by the police, or in the hiring process. Experiencing racial discrimination from the police was associated with the greatest increase in odds of food insecurity at 11.76 (1.41, 97.86) at *p* < 0.05, when compared to those who did not experience racial discrimination. However the increased odds of food insecurity associated with experiencing racial discrimination in the housing process (OR: 7.9, 95% CI: 1.93, 32.34) and in the hiring process (OR: 6.81, 95% CI: 1.98, 23.48) reached greater statistical significance (*P* < 0.01).

**Table 2:** Discrimination Factor and odds of food insecurity

	n (%)	Food Insecurity Odds Ratio (95% CI) Unadjusted Model	Food Insecurity Odds Ratio (95% CI) Adjusted Model
**Location of discrimination**			
At Work			
No	91 (63.64)	Ref	Ref
Yes	52 (36.36)	1.96 (0.92, 4.19)	1.77 (0.79, 3.98)
**At School**			
No	91 (63.64)	Ref	Ref
Yes	52 (36.36)	2.67 (1.21, 5.88)*	2.81 (1.20, 6.61)*
**Housing**			
No	120 (83.92)	Ref	Ref
Yes	23 (16.08)	6.76 (1.51, 30.34)*	7.90 (1.93, 32.34)**
**Medical**			
No	115 (80.42)	Ref	Ref
Yes	28 (19.58)	3.86 (1.25, 11.90)*	3.95 (1.07, 14.67)*
**Restaurant**			
No	86 (60.14)	Ref	Ref
Yes	57 (39.86)	2.11 (1.00, 4.43)*	2.04 (0.83, 5.02)
**Loans**			
No	123 (86.01)	Ref	Ref
Yes	20 (13.99)	5.57 (1.23, 25.21)*	3.82 (0.71, 20.53)
**On the street**			
No	87 (60.84)	Ref	Ref
Yes	56 (39.16)	2.71 (1.26, 5.86)*	3.01 (1.23, 7.34)*
**By Police**			
No	119 (83.22)	Ref	Ref
Yes	24 (16.78)	15.55 (2.02, 119.88)**	11.76 (1.41, 97.86)*
**In Hiring**			
No	104 (72.73)	Ref	Ref
Yes	39 (27.27)	4.99 (1.80, 13.83)**	6.81 (1.98, 23.48)**

Adjusted models controlled for race, gender, age and income. Individual discrimination components’’ relationship with food insecurity was assessed with logistic models at p-values of *P < 0.05, **P < 0.01, ***P < 0.001

Table 3 shows the associations between discrimination and stress, physical health, food insecurity and grade point average. When measured continuously, racial discrimination scores were negatively associated with physical health, stress and food security. However, racial discrimination was positively associated with grade point average. When discrimination was measured as a binary variable, only the negative associations between racial discrimination and stress and racial discrimination and food security persisted. A one unit increase on the discrimination scale was associated with a 0.02 (0.01, 0.04) greater stress score. A one unit increase in racial discrimination scale was associated with a 1.18 (1.06, 1.32) greater odds of experiencing food insecurity.

**Table 3:** Association between Discrimination and Physical health, Stress, Food Insecurity and Grade Point Average

How discrimination was measured	Physical Health Coef	95% CI	Stress Coef	95% CI	Grade Point Average Proportional Odds Ratio	95% CI	Food Insecurity Odds Ratio	95% CI
Discrimination measured as a continuous variable	0.03	(0.01, 0.05)**	0.02	(0.01, 0.04)**	0.95	(0.91, 1.00)*	1.18	(1.06, 1.32)**
Discrimination measured as a binary variable	0.25	(-0.03, 0.53)	0.26	(0.06, 0.46)*	0.46	(0.20, 1.05)	3.76	(1.55, 9.10)**

All models controlled for race, gender, age and income. Linear models assess discrimination and stress and physical health. Ordered logistic models assessed discrimination and grade point average where the baseline was those reporting an A or A- GPA at p-values of *P < 0.05, **P < 0.01, ***P < 0.001

## Discussion

Average composite racial discrimination scores were lowest among White students, but only those who were Asian (not including Chinese, Filipino, and Asian Indian), and Other Race were significantly higher. Interpreting these results are challenging due to the potential heterogeneity of these groups, however higher discrimination scores have been associated with an array of negative health effects, including mental health outcomes [28], eating competence [29], poor sleep [30] and cancer [31]. Consistent with past research, we observed a statistically higher amount of racial discrimination among those who were food insecure compared to their food secure counterparts.

Racial discrimination across all constructs except at work were significantly associated with greater odds of food insecurity in the unadjusted models. However, when adjusting for race, gender, age, and income, racial discrimination was positively associated with a greater odds of food insecurity when experienced at school, housing, medical, on the street, by police/courts, and in the hiring process. The largest association was seen when students had perceived racial discrimination when dealing with the police and court system, where the odds ratio was 11.76 (1.41, 97.86). This is consistent with the work of Phojanakong et al., who found that discrimination from police/courts and in workplaces was positively associated with food insecurity while this was not the case for discrimination experienced elsewhere [16]. Racial discrimination in the court system may increase the likelihood of incarceration, or penalties and fines assessed, all of which could directly impact food insecurity. The immediate link between racial discrimination perpetuated by police and students that leads to food insecurity is less clear, although being unfairly treated by police may increase stigma dealing with government and assessing food assistance programs. The other two strongest links observed between racial discrimination and insecurity were observed when the discrimination occurred in the hiring process, or in the housing process. Clearly, if you are discriminated against in the hiring process this reduces your ability to procure the job, and to negotiate your salary, both of which clearly impact your financial status which is tightly linked with food insecurity. Similarly, if you cannot acquire a room to rent or can procure an affordable place to live, this could impact your disposable income or the likelihood you become unhoused, both of which would likely impact your food security status. Qualitative research or more in-depth quantitative research is needed to help elucidate the connections between experiencing racial discrimination experienced on the street, accessing loans, in a restaurant etc. and food security status.

Finding that racial discrimination was positively associated with GPA was unexpected, and is contrary to previous studies [4–6]. However, there have been other studies with similar findings that cite student’s resilience, indicating that sometimes students who face discrimination may be more driven or focused on academic achievement than their peers not facing racial discrimination [32, 33]. In 2017 Fuller found among black athletes that there may be a tipping point between stereotype management and stereotype threat, where beyond a threshold of perceived racial discrimination students’ academic performance is hindered [33]. Owens’ work highlights the important role of immigration status, where students of color who are recent immigrants are less susceptible to stereotype threats than second generation immigrants [32]. Future work on intersectionality may present a clearer picture to competing phenomenons that may better explain the complex relationship between racial discrimination and academic performance.

Consistent with past research, composite racial discrimination was negatively associated with physical health and stress [2, 3]. Experiencing any discrimination was associated with a 0.25 (-0.03, 0.53) average lower physical health score, and self reported health questions such as those implemented in this study have long been connected with various negative health outcomes such as cancer, diabetes and other diseases [34]. Similarly, a 0.26 (0.06, 0.46) greater average stress score across the 10 questions is an overall increase of 2.6 in the score. Given scores of 16–20 are associated with a high health concern and scores of 21 + are associated with very high health concern and this population had an average score of 21.4 already, this change in 2.6 could shift students across levels of concern [35].

### Limitations

There are several limitations of this study. Race is a socially constructed variable that is imperfectly measured, having groups such as Other Asian, and mixed race, suggest we have likely categorized individuals who have heterogeneous experiences with racism and may represent a myriad of populations. Additionally, our small sample of Black students had to be included in our “other” group, which may have led to inadequate adjustment for race given research suggests Black students experience relatively large amounts of racial discrimination compared to other racial and ethnic groups [33]. Race and grade point average were self-reported, which made lead to misclassification bias. The small sample size leads to larger confidence intervals which may be impeding our ability to ascertain statistically significant findings, meaning the absence of significant associations here do not provide much evidence that one does not exist. This study design is cross sectional, which limits our ability to infer causality because we cannot establish temporality, and there is potential for reverse causality.

Therefore, significant associations found in this study should not be considered causal. We also did not gather information on participation in food assistance programs or campus food pantry use, which would have helped us interpret if racial discrimination acts through food assistance participation to impact food insecurity.Finally, data was collected post Covid-19 pandemic which could affect students' experiences.

## Conclusion

The location in which a student experienced racial discrimination impacted the extent to which the racial discrimination was associated with food security status. Although our sample was small, the associations between discrimination experienced by the police and while accessing housing and food insecurity were very large, and warrant further exploration. While this finding is novel amongst college students, to better explore the relationships between racial discrimination and food insecurity and other health and academic outcomes, a larger population with more racial groups that could be studied over time is needed to allow for greater generalizability and to identify causal relationships. Further research is needed to explore potential mechanisms for how racial discrimination may lead to food insecurity.

## Data Availability

The datasets used and analyzed during the current study are available from the corresponding author on reasonable request.
