# Correction: Racial discrimination is associated with food insecurity, stress, and worse physical health among college students: Lana Wood

**DOI:** 10.1186/s12889-024-18521-x

**Published:** 2024-04-15

**Authors:** Ryan Gamba, Negin Toosi, Lana Wood, Alexandra Correia, Nomar Medina, Maria Pritchard, Jhamon Venerable, Mikayla Lee, Joshua Kier Adrian Santillan

**Affiliations:** 1 Department of Public Health, California State University, East Bay. SF 102. 25800 Carlos Bee Boulevard, 94542 Hayward, CA USA; 2 Department of Psychology, California State University, East Bay. 25800 Carlos Bee Boulevard, 94542 Hayward, CA USA; 3 University Libraries, California State University, 94542 East Bay, Hayward, USA

**Correction: BMC Public Health (2024) 24:883**

10.1186/s12889-024-18240-3

The original publication of this article contained 2 incorrect author names. The incorrect and correct information is listed in this correction article. The original article has been updated.

Incorrect

Venerable Jhamon

Correia Alexandra

Correct

Jhamon Venerable

Alexandra Correia

